# *“We shall have gone to a higher standard*”: Training village heath teams (VHTs) to use a smartphone-guided intervention to link older Ugandans with hypertension and diabetes to care

**DOI:** 10.12688/aasopenres.13049.1

**Published:** 2020-06-23

**Authors:** Joseph Okello Mugisha, Janet Seeley

**Affiliations:** 1 Medical Research Council/Uganda Virus Research Institute and London School of Hygiene and Tropical Medicine, Uganda Research Unit, P.O.Box 49, Entebbe, Uganda; 2 Department of Global Health, London School of Hygiene & Tropical Medicine, Keppel Street, London, WC1E 7HT, UK

**Keywords:** ageing, non-communicable diseases, community health workers, Uganda

## Abstract

**Background**: It is not clear whether village health teams (VHTs) can be empowered to participate in interventions to prevent and control hypertension and diabetes in older adults in Uganda. We conducted this study in rural Uganda to establish the experiences of VHTs in managing older adults with health problems, their knowledge of hypertension and diabetes and their understanding of referral systems. We also explored their experiences with smartphones and whether VHTs could be effectively trained to use a smartphone-guided intervention to link older adults with hypertension and diabetes mellitus to care.

**Methods:** We conducted in-depth interviews (IDIs) with and trained 20 VHTs randomly selected from Bukulula sub-county in Kalungu district from October 2017-December 2018. We used interview guides to explore topics relevant to our study objectives. VHTs were trained to measure blood sugar and blood pressure using digital machines. VHTs were trained on identifying symptoms of diabetes mellitus. Data from IDIs were analysed using thematic content analysis. Competence tests were used to evaluate the training.

**Results:** Most of the VHTs were female (75%). All VHTs had some knowledge on hypertension and diabetes and other chronic diseases. They did not have any experience in treating older adults since they had been trained to deal mainly with children. Half of the VHTs owned smartphones. All were willing to participate in an intervention using a smartphone to link older adults with hypertension and diabetes mellitus to care. By the end of the training, all but three participants could comprehend the symptoms of diabetes and measure blood sugar and blood pressure.

**Conclusion: **Village health teams in the study setting need training in managing the health needs of older adults before engaging with an intervention using smartphones to link older adults with diabetes mellitus and hypertension to care.

## Introduction

The number of older adults living with chronic conditions is increasing rapidly in Uganda ^1– 4^. These chronic conditions include hypertension, diabetes mellitus, chronic obstructive pulmonary disease, cardiovascular diseases, osteoarthritis and cancer. Evidence available suggests that most of these older people with chronic conditions either do not know that they have them ^3, 5, 6^, or may not be on treatment ^7^.

Recently, greater attention has been paid to interventions for the prevention and control of chronic conditions in public health care ^8– 10^. However, in sub-Saharan Africa these interventions do not usually focus on older people, who are disproportionately affected by chronic conditions ^11^. Studies conducted in Uganda and elsewhere in Africa have shown that older adults have delayed access to health care due to a number of reasons ^12– 14^. These may include personal factors like poor health, lack of resources and, for chronic diseases, awareness of disease symptoms. Others may include factors related to the health care facilities, including responsiveness of the health care staff to older people and availability of the drugs.

With the increasing numbers of older adults with chronic conditions, especially hypertension and diabetes mellitus, there is a need for public health interventions that will improve referral of older adults with chronic conditions from within their communities to formal health care facilities for proper diagnosis and treatment. One of the possible public health interventions that could be used is to strengthen the capacity of VHTs to screen older adults in their communities for some of the more prevalent chronic conditions like hypertension and diabetes. Once screened, those suspected to have chronic conditions can be referred to health care facilities for full diagnosis and treatment.

Village health teams (VHTs) are community health workers in Uganda. The concept of these VHTs was introduced in Uganda by the Ministry of Health to promote health services, including referral of people to formal health care facilities ^15^. The VHTs are selected by their communities, who agree on who they want to represent them. The VHTs are supposed to work on a voluntary basis, although a small facilitation payment is given depending on the intervention. Some of the responsibilities of the VHTs include mobilising the communities for health action, promoting health and preventing diseases, treating simple illnesses at home, checking for danger signs at home, referring sick people to health workers and keeping up-to-date village records ^15– 18^.

The VHTs are at the lowest end (village level) of the Ugandan health care system, which starts with the Ministry of Health, national referral hospitals, regional referral hospitals, district hospitals, health centres at level four (constituency level), health centres at level 3 (sub-county level), health centres at level 2 (parish level) and then VHTs ^19^. 

There are some advantages of involving VHTs in the control and prevention of chronic conditions in Uganda. First, VHTs are already part of the primary health care system in Uganda and have been involved in a number of interventions, especially in maternal and child health. Second, the health care system in Uganda is understaffed and VHTs could play a role in the screening of older adults with chronic conditions. In addition, VHTs work on a voluntary basis and thus few resources are needed for the VHTs to be involved in the prevention and control of chronic conditions.

The use of smartphones in rehabilitation, linking of patients to care and management of patients is now increasing ^20– 24^. In Uganda, it has been shown that community health workers can use smartphones to register pregnant women at home and relay gestation age-specific SMS messages to them to reduce home births ^25^. A study in Uganda looking at the experiences of using mobile phones in everyday life among people who had suffered a stroke and their families established that mobile phones can play a key role in the rehabilitation of patients ^26– 28^. 

In preparation for developing a smartphone-guided intervention to strengthen the capacity of VHTs to screen and refer older adults with hypertension and diabetes to care, we conducted this preliminary study among VHT members from Kalungu district in rural southwest Uganda to establish the experiences of VHTs in dealing with older people with hypertension and diabetes mellitus, their experiences with smartphones and to establish whether VHTs were willing and could be effectively trained to use a future planned smartphone-guided intervention to link older Ugandans with hypertension and diabetes mellitus to care.

## Methods

### Ethical statement

The Uganda Virus Research Institute Research and Ethics Committee (GC/127/18/01/630) and the Uganda National Council for Science and Technology approved this study (SS4530). Before the interviews, a study information sheet was administered to all study participants and all those that agreed to participate signed a consent form. In addition, permission to audio record the interviews was sought from all the VHT members that were interviewed.

### Study setting

This study was conducted in Bukulula sub-county, Kalungu district in rural southwest Uganda between October 2017 and December 2018. According to figures from the Uganda Bureau of statistics, the population of Kalungu district was approximately 350,000 in 2017 and 5% of this population were aged 60 years and above ^29^. Bukulula is the second most populous sub-county in the district and lies along the highway that connects Kampala to Tanzania, Rwanda and the Democratic Republic of Congo. The population of Bukulula sub-county depend on agriculture and migrant labour for their livelihoods but some communities around the shores of Lake Victoria depend on fishing. Within Kalungu district, 41% of the females aged 10 years and above own mobile phones, compared to 44.8% of the males aged 10 years and above. The proportion of the population with smartphones is not known. The district has two level 4 health centres, with one of these health centres located at Bukulula sub-county headquarters. There are a number of other public and private health facilities within the district that cater for both communicable and non-communicable diseases.

### Study design

The study was done in two phases. The methodological orientation we used in phase one of this study was content analysis. In phase 1, we used qualitative research methods to collect data from the selected VHT members. During the second phase, we conducted training sessions with all the 20 VHT members that had participated in phase one. During the training, we used short presentations, discussions and practical exercises that involved all the selected VHT members.

### Study population

We selected 20 members of the VHTs from within Bukulula sub-county. Since this was a preliminary study in preparation of a bigger study in future, a sample size of 20VHT members would be able to provide us with enough qualitative information that we needed to answer our research question. These were randomly selected from all the VHTs in the sub-county. All the names of the 138 VHTs within the sub-county were written on papers and churned in a container. The District VHT Coordinator used a raffle draw method until all the 20 VHTs were selected. The inclusion criteria were that the VHT had to be a resident of Bukulula sub-county and should have been active as a VHT within the sub-county in the last three months. VHTs were excluded if they were not in a position to participate in the interviews and were not willing to attend the training. The selection of the VHTs was done in consultation with stakeholders within the sub-county and the district including the coordinator of the VHTs within the district. Bukulula sub-county has 69 villages and each village has two VHT members that had been previously recruited following the Uganda Ministry of Health guidelines for selection of VHTs ^16^. Out of the twenty originally selected, two were replaced because they declined to participate. They declined participation because they were working from far away in Kampala (capital city of Uganda) and did not have time to come back to Bukulula to participate in the interviews. 

### In-depth interviews (IDIs)

Before IDIs were conducted, all the selected VHT members were invited for a meeting (through a phone call made by the District VHT Coordinator), which was co-chaired by the principal investigator (PI) and the district VHT coordinator. This meeting was conducted at Bukulula Health Centre Level 4. During the meeting, we explained all study procedures to the selected VHT members and a schedule for the interviews was made and agreed to by all the selected VHT members.

The IDIs were conducted by the PI (JOM) together with a female social science research assistant. The research assistant completed high school but later trained as a social science research assistant and has been working as a social sciences research assistant with MRC/UVRI and LSHTM Uganda Research Unit for a period of 25 years. Interviews were conducted in English (for VHTs who were comfortable with English) and in Luganda, the local language used in the study setting (for those who preferred Luganda). The interview duration was 45 minutes to one hour. All interviews were conducted in a quiet room at Bukulula Health Centre in the morning. The interviews followed an interview guide that explored the following: demographic characteristics; VHT recruitment; training and activities of the VHTs; experiences in dealing with older people; linking of people with health issues to care; knowledge of common chronic diseases; experiences and perception of smartphones; and willingness to use a smartphone-guided intervention in the future to link older people with chronic conditions to care. All interviews were audio-recorded and field notes made.

### Training of VHTs

The VHTs underwent three-day non-residential training. The training was basic given the level of education of the VHT members (very few had completed ordinary level at senior school, despite Uganda Ministry of Health recommendations that VHT members should be educated to this level). The training sessions were conducted by the PI (JOM), the district VHT coordinator and a qualified nurse. During the training, the following topics were covered: ageing in Uganda and some common diseases associated with ageing; introduction to hypertension and diabetes mellitus; identifying simple symptoms and signs of hypertension and diabetes mellitus; complications of hypertension and diabetes mellitus; the referral process from villages to formal health care facilities and what should be included on referral forms. VHT members were also taught how to draw blood and test for blood sugar using a portable glucometer (ONETOUCH SelectSimple blood glucose meter) ^30^, and how to measure blood pressure using a digital blood pressure machine (OMRON, Automatic Upper Arm Blood Pressure Monitor M7 Intelli IT (HEM-7322T-E)) ^31^. In addition, practical exercises for measuring blood sugar and measuring blood pressure were conducted during the training sessions. A qualified nurse, working with MRC/UVRI and LSHTM Uganda Research Unit and the District VHT Coordinator, who is a qualified clinical officer, conducted the practical sessions. The practical sessions were supervised by the PI (JOM), who is a qualified medical doctor with a long-term experience in managing older people with chronic conditions in Uganda. Each VHT member drew blood from a colleague and tested the blood sugar level. Likewise, each VHT member took at least three blood pressure measurements from a colleague and recorded the blood pressure measurements. All the practical exercises were conducted in the sub-county hall at Bukulula sub-county headquarters near Bukulula Health Centre Level 4.

### Data management and analysis

Transcription and translation (for interviews conducted in Luganda) of the interviews was undertaken by two social science research assistants within two days after the interviews. After the first two interviews were transcribed and translated, the PI and the social science research assistant involved in the data collection read the scripts to check for completeness of data collection. After the first two interviews, the interview guide was slightly revised. The modifications that were made after the two interviews were introducing a question to capture data on the challenges faced by VHTs while doing their work. When all the interviews were completed and after reading through all the interview scripts and exploring the emerging themes, the PI and the social science research assistant agreed on a coding framework that was used to code all the data. To code the data, we used a matrix with the main themes that arose from the data including nomination procedures for becoming VHT, period of service as VHT, challenges faced by VHTs, definition of an older person, definition of chronic conditions, dealing with older people with chronic conditions, experiences with smart phones, ownership of smart phones, acceptability of using smart phones in a future intervention and problems likely to arise with use of smart phones. Data were analysed by thematic content analysis.

For training, competence tests were used to evaluate the practical exercises. Competency scores were given for all steps of the practical exercises. For practical exercises, one point was awarded for each step done correctly. The final score was calculated in percentages. Tests for blood pressure measurement and blood sugar measurement were scored separately. For blood sugar measurement, these steps included:

1. Preparing the person for the blood draw
2. Wearing gloves correctly
3. Inserting the strip in the glucometer correctly
4. Identifying and cleaning the finger for the blood draw
5. The procedure of drawing blood
6. Reading the glucometer correctly
7. Removing the gloves correctly
8. Proper disposal of sharps and other materials
9. Recording an accurate reading from the glucometer
10. Final advice given to the client in accordance with the level of the blood sugar

For blood pressure measurement, competency scores were given for the following steps:

1. Preparing the person for blood pressure measurement
2. Using the right blood pressure cuff
3. The procedure of measuring the blood pressure
4. Accurate recording of the blood pressure measured
5. Advice given to client after blood pressure measurement

If a VHT member scored 80% and above for these steps, we considered them to have passed the competence test.

## Results

### Demographic characteristics of study population

In total, we performed IDIs with 20 VHTs. Most of the VHT members were female, 16 (80%), and married, 16 (80%), and only six (30%) had completed ordinary level in secondary school (locally known as senior 4). The sample included four (20%) VHT members who were aged 60 years and over. The majority of these VHT members were mainly small-scale farmers, 15 (75%), and had been serving as VHT members for five years and over, 15 (75%).

After obtaining demographic information from the study participants, we went on to establish their experiences in dealing with older adults.

### Experiences in dealing with older adults with health issues

We first asked the participants how they defined an older person or how they knew a person was old. Most of the study participants defined ageing according to physical appearance and rate of work. 

*You understand that someone is aged depending on how she/he appears because you may find someone and notice she has become weak/reduced strengths and when you look into the face, you realise she is aged. She could be having grey hair, you can realise she is weak in what she is doing, has reduced strengths and when you merely look in her face, she is aged.* (40–50-year-old female VHT, primary school education).

When this same participant was asked about age, she replied with the following:

*In most cases, majority* [referring to older adults] *do not tell their age/true age yet others do no happen to know. However, if you happen to have followed age, personally I can start from 50 years onwards. That person happens to be old*.

When another respondent was asked how you know that someone was an older person, she said:

*She would be starting from the age of 60 years, then you can know that she is old and okay, another condition is bending of the back, when she ages, she has signs she shows. She moves with the support of the stick. Some cannot support themselves. Sincerely, the work she used to do at the young age, she cannot manage at the moment* (30–40-year-old female VHT, secondary school education)

When another participant was asked whom he considers an older person to be, he said:

*He would have been…Ha…you see I am also an elderly. Let us say ageing comes about or takes place at every stage. You might be 50 but then you become old; or when you fall and the back becomes affected and you are like this….* [He demonstrated moving with his back curved]. *Or when you get an accident and you no longer have any work to do* (60–70-year-old male VHT secondary school education)

None of the VHT members had experience in treating old people with health problems. It was noted that when the concept of VHTs was introduced in Kalungu district, they were selected and trained to only deal with children between two and five years who had fever and diarrhoea. At some point, other maternal and child health programmes were added to their responsibilities. However, some VHTs assist in supervising people with chronic conditions like tuberculosis (TB) who are referred to them from formal health care facilities for supervision in taking their medications properly. 

*I also see some elderlies* [older people] *because I usually treat some elderlies who have TB. They usually refer them to me, those men who are found with TB; they smoke cigarettes and tobacco so much. I have still treated them and at times, they go to health centres* (40–50-year-old female VHT, secondary education).

This same VHT member went on to say that she also helps monitor adherence for patients on TB treatment:

*You happen to be there and they phone you* [phone call from health centre] *, VHT such and such a patient does not swallow/take tablets well. There is even a health worker who came just looking for him, he got him and then handed him* [patient] *to me. My dear God blessed me, whenever he used to bring the tablets they* [health workers] *would tell him, take them to the lady* [VHT member]. He had stopped taking the tablets for about a year and then they started him on the drugs afresh. I then started administering it to him.

### Knowledge of chronic conditions

All the VHT members were able to define and mention some of the chronic conditions that were common in their communities. Some of the diseases mentioned included hypertension, diabetes, asthma, HIV/AIDS, diseases of the heart, cancer, prostate cancer, persistent cough, stomach ulcers, swelling of the whole body, and possession of cholesterol. However, their understanding of the impact of these diseases for an individual, their family and the community was mixed. While all of them understood the impact of the chronic diseases to an individual, half of them did not understand the impact of chronic diseases on the family and at the community level.

When one VHT was asked to mention some of the chronic diseases that affect older people, he said

*I can understand like the HIV, say like these illnesses…what are they called? HIV, I have talked about it; the chronic diseases, sometimes you might…only that even diabetes we count it among them. You might fall sick with Asthma, you can fall sick with; what do they call it? Someone being that he experiences swelling of his body. They have these illnesses those that somehow old; experience frequent urination; within that old age. It is to say that when a man or a woman, has to pad himself/ herself. Yes, such illnesses* (30–40-year-old male, secondary education).

When asked about chronic diseases that affect older people, another VHT said

*Those sicknesses, backache, legs (meaning painful legs), pressure/hypertension, diabetes; all diseases collect when one becomes old; poor eye sight, mist like substances within the eyes; things of that kind/such diseases* (60–70-year-old male, secondary education).

### Experiences of VHT members with smartphones

All VHT members had seen and some owned smartphones. A big proportion (70%) of the VHTs did not know a local name for the smart phones but others gave local names including *koloboza* (putting a finger and scrolling), *kuseereza* (scrolling), *kukoona* (to hit) *, songa* (starting), *and kibaati* (made of iron). These are local names in the Luganda language. Most of these words refer to how smart phones are used by placing a finger on the screen of the smart phone.

VHT members reported the functions of smart phones as: making and receiving calls, Facebook, WhatsApp, Twitter, taking and sending messages, taking and sending photographs, making processes quicker, enabling owners to become dignified (being held in high esteem within the community because of using smart phones), recording voices, having access to voicemails, taking records/documents and knowing what is happening in the international world easily by connecting to the internet.

When we asked them if they knew of any government programme using smartphones, three participants mentioned the Coffee Development Authority (CDA) programme that gave smartphones to some selected farmers in their communities and that these farmers were sending information to the secretariat of the CDA. However, they did not know the type of information that was being sent. Another participant talked of a small-scale loan organisation called BRAC.

All the VHT members were interested in the use of the phones and said they would accept using a smartphone-guided intervention to link older people with chronic conditions to care.

*“We shall be more than happy to use these phones to send old patients to health centres. We shall feel good. We shall have gone to a higher standard. And it becomes easy for us because I happen to be referring a patient quickly and easily….”* (60–70-year-old male, secondary school education).

“I shall be very pleased to use those phones [smartphones] *. I shall be very pleased health worker* [referring to interviewer] *because I had earlier longed to use that phone before but not able to buy it. However, longing for it when you happen to give it to me free of charge, I can become very happy to use it in that program you talked about of older people”* (40–50-year-old female, completed primary 7)

Although most of the VHT members were willing and happy to use smartphones to link older people with chronic conditions to care, they expressed a number of challenges they would face while using these smartphones, including the need for rigorous training to learn how to use them, theft of smartphones and failure to find somewhere to charge the phone (since the reach of the electricity supply in the area is limited).

*“It would be when I have not yet learnt to use I* [smartphone]. *You can give it to me doctor but when I do not know how to use it. That is one problem/challenge. Another problem it has these* [smartphones] *are very much liked by the kyala kimpadde* [thugs] *. Then in case it does not have power, I do not have electricity at my home; I have to take it where….at the shop so that they do what….they might steal it from there. That is a big challenge reporting to my boss that they have stolen the phone from me! At times he might not have trust in me, can say you are lying, you sold it. Now that problem is there when it happens to have used up power. It is a big problem* (40–50-year-old female, secondary education).

### Practical exercises

All VHT members said the practical exercises were very exciting and expressed their wish that it would be good if they could have these frequently during their routine work. Most of them were competent with finger pricks, probably because they do finger pricks frequently when testing children for malaria. Three participants out of 20 (15%) failed the competence test for the practical exercises.

## Discussion

Most of the VHT members in this study had not attended secondary school, contrary to the guidelines of the Ministry of Health of Uganda that recommend that all VHT members should have a minimum qualification of ordinary level (senior school). None of the VHT members had experience in dealing with older people with chronic conditions, since their initial training concentrated on treating or referring children with fever within their communities. All VHT members had knowledge of the common chronic conditions like hypertension and diabetes that affect older people within their communities. Smartphones were widely used within the study setting and a number of the VHT members owned smartphones. The acceptability of using a smartphone intervention for linking older people with chronic conditions to care in future was high among VHT members. We also established that VHT members could be effectively trained to measure blood sugar and blood pressure using digital machines. 

The use of community health workers has been an ongoing practice, especially in their contribution towards child survival ^32– 35^. These community health workers have been undertaking various tasks including case management of malaria, diarrhoeal diseases, pneumonia and neonatal sepsis ^34, 36^. In addition, community health workers have played a role in other areas of maternal and child health, health promotion and linking communities to formal health care ^15^.

With an increasing burden of chronic conditions in Uganda, there is need to start exploring ways in which VHTs can contribute to the prevention and control of chronic conditions, especially among older people, who are disproportionately affected by chronic conditions and at the same time have low access to health care facilities ^13, 37^. Since VHTs stay in the communities where older people with chronic conditions live, they can participate in health education talks about prevention of chronic conditions, screen people for chronic conditions and link them to formal health care facilities. In addition, they could also play a role in monitoring adherence to medication for chronic conditions. There will be a need to first train VHTs on the ageing process and generally dealing with older people who have health issues within their communities. In a recent systematic review conducted in low and middle income countries (LMICs), it was shown that compared with standard care, using community health workers in health programmes has the potential to be effective in LMICs, particularly in tobacco cessation, hypertension and diabetes control ^38^.

The use of smartphones in Uganda is increasingly being adopted by the public. Although the exact proportion of Ugandans using smartphones is not clearly known, a good number of general public and health professionals use them for various reasons. However, they have not widely been used in health programs in Uganda. The smartphone applications can be used at different points in health care including prevention, linkage to care, diagnosis, prescription, patient self-management and rehabilitation ^21^. The availability of smartphones for the public and the willingness to use them by VHT members in this study makes them suitable to use in a future intervention to link older people with hypertension and diabetes to care. A study conducted in Uganda looking at the acceptability and perceptions of community health workers on mHealth in the field of HIV/AIDS established that there was enthusiasm for mHealth and the method was acceptable to community health workers ^39^. Another study conducted in a similar study setting in Kalungu district established that community health workers could effectively use smartphones to register pregnancies and birth outcomes ^25^.

In this study, a number of inhibiting factors like theft, storage, charging of the smartphones and training were mentioned. These will need to be addressed before an intervention using smartphones to link older people with hypertension and diabetes to care is rolled out.

Our findings show that VHTs in Uganda can be effectively trained and are enthusiastic about an intervention using smartphone applications to link older people with hypertension and diabetes to care. We did not train older people themselves to see if they are willing and able to use a smartphone-guided intervention to self-link to care. Considering the literacy and education level of older people in Uganda, we believed that this was not feasible among this age group. However, we believe that this could be feasible in younger adults who may be able to use such an intervention to self-link to health care facilities in the case of chronic conditions.

In conclusion, VHTs in rural southwest Ugandan will need training in dealing with older people with health problems before they get involved in an intervention using a smartphone application to link older adults with chronic conditions to care.

## Data availability

### Underlying data

LSHTM Data Compass: Data for: Feasibility of village health teams (VHTs) in using a smart phone guided intervention to link older Ugandans with chronic conditions to care. https://doi.org/10.17037/DATA.00001699

This DOI will remain accessible in the long-term. If LSHTM ceases to operate or the data repository is replaced with another system, arrangements would be made to redirect this DOI to its new location. Researchers interested in obtaining further details on the transcripts are asked to complete the request form operated by the repository. The request form provided to contact the researchers will be maintained for the foreseeable future. Submitted forms should be emailed to Joseph Mugisha ( joseph.mugisha@mrcuganda.org) and Janet Seeley ( Janet.seeley@mrcuganda.org) for follow-up, as well as sent to the repository administrator who maintains the system. Contact emails can be updated at a later date as required, e.g. in the event that project contacts move to another institution, a new project contact is defined, the institution's name is changed, etc. The transcripts cannot be made openly available for data protection and ethical reasons. Participants are not named in transcripts, but there are sufficient contextual details to allow indirect identification. The consent agreement signed by participants also sets conditions that limit access to authorised people within the study team only. Following receipt of a data request, Dr. Mugisha & Dr. Seeley would enter into a dialogue with the requester to answer their questions (with advice provided by the research data manager, as required). The project will be unable to make the full un-anonymised transcript available, however selected non-identifiable text might be provided to clarify specific points.

### Extended data

LSHTM Data Compass: Data for: Feasibility of village health teams (VHTs) in using a smart phone guided intervention to link older Ugandans with chronic conditions to care. https://doi.org/10.17037/DATA.00001699

- VHT_study_Consent_form_English.pdf (Information sheet and consent form)
- VHTs_Smartphone_FeasibilityStudy_InterviewGuide.pdf (Interview guide for study investigating feasibility of village health teams (VHTs) using smartphone-guided intervention to link older Ugandans with chronic conditions to care)

Data are available under the terms of the Creative Commons Attribution 4.0 International license (CC-BY 4.0).
