# Supplementary material for: “We shall have gone to a higher standard”: Training village heath teams (VHTs) to use a smartphone-guided intervention to link older Ugandans with hypertension and diabetes to care
Source: AAS Open Res. 2021 Dec 1;3:25. Originally published 2020 Jun 23. [Version 2] doi: 10.12688/aasopenres.13049.2 (PMC8729021; doi:10.12688/aasopenres.13049.2)
Supplement: Peer review history

# Peer review: Reviewer response for version 2

**DOI:** 10.21956/aasopenres.14418.r29022

**Published:** 2021-12-22

**Authors:** Gerald A. Paccione, Kristen Welch

**Affiliations:** 1 Montefiore Medical Center, Bronx, New York, NY, USA; 2 Albert Einstein College of Medicine, New York, NY, USA

We have re-reviewed the manuscript, and find it has successfully addressed most of our questions/issues previously raised. It's a bit redundant in places (e.g. intro and methods descriptions of who the VHTs are), but I/we can approve it for indexing.

Is the work clearly and accurately presented and does it cite the current literature?

Partly

If applicable, is the statistical analysis and its interpretation appropriate?

Not applicable

Are all the source data underlying the results available to ensure full reproducibility?

Partly

Is the study design appropriate and is the work technically sound?

Yes

Are the conclusions drawn adequately supported by the results?

Yes

Are sufficient details of methods and analysis provided to allow replication by others?

Partly

Reviewer Expertise:

Global Health, program implementation in Africa. General Internal Medicine.

We confirm that we have read this submission and believe that we have an appropriate level of expertise to confirm that it is of an acceptable scientific standard.

# Peer review: Reviewer response for version 2

**DOI:** 10.21956/aasopenres.14418.r29023

**Published:** 2021-12-13

**Authors:** Dorothy Lall

**Affiliations:** 1 Institute of Public Health, Bengaluru, Karnataka, India; 2 Christian Medical College and Hospital, Vellore, Tamil Nadu, India

No further comments to make.

Is the work clearly and accurately presented and does it cite the current literature?

Partly

If applicable, is the statistical analysis and its interpretation appropriate?

Not applicable

Are all the source data underlying the results available to ensure full reproducibility?

Partly

Is the study design appropriate and is the work technically sound?

Partly

Are the conclusions drawn adequately supported by the results?

Partly

Are sufficient details of methods and analysis provided to allow replication by others?

Yes

Reviewer Expertise:

Primary health care, Non communicable disease control, organisation of chronic care, chronic care models, person centred care, community based models

I confirm that I have read this submission and believe that I have an appropriate level of expertise to confirm that it is of an acceptable scientific standard.

# Peer review: Reviewer response for version 1

**DOI:** 10.21956/aasopenres.14144.r28343

**Published:** 2021-03-15

**Authors:** Dorothy Lall

**Affiliations:** 1 Institute of Public Health, Bengaluru, Karnataka, India; 2 Christian Medical College and Hospital, Vellore, Tamil Nadu, India

This manuscript describes VHTs perspective on being included in the team that manages chronic conditions. While the manuscript is written clearly, there are a few methodological gaps I would like to highlight that I hope will will enable the authors to refine this report. 

1. The research question is not clear - it can be stated as an exploratory question to understand VHT experience and willingness to care for elderly with chronic conditions using a smart phone.
2. The methods stated are qualitative however, the sampling described is a probability sampling method - the two are not coherent. A purposive sample with a mix of men and women (if that is contextual), old and young, differing levels of education, social position, would have been appropriate to answer the question.
3. The analysis described uses the term thematic content analysis - either this is content analysis or it is thematic.
4. The analytic process described begins with themes - however in content analysis there is no use of themes and in thematic analysis it is the end result of a coding process. 
5. The results for the experiences in dealing with older adults and knowledge of chronic conditions are stated as individual narratives however the analysis should have enabled a general theme if thematic analysis or if content analysis.
6. A table describing the participants would be useful to understand variation in response
7. Only practical competency for testing blood glucose and blood pressure was measured. Identification of whom to screen, when to refer and other programmatic elements including use of smartphone was not assessed. Hence to conclude that VHTs can be effectively trained is an overstatement. To conclude about effectiveness of training before and after comparison would be required.

Is the work clearly and accurately presented and does it cite the current literature?

Partly

If applicable, is the statistical analysis and its interpretation appropriate?

Not applicable

Are all the source data underlying the results available to ensure full reproducibility?

Partly

Is the study design appropriate and is the work technically sound?

Partly

Are the conclusions drawn adequately supported by the results?

Partly

Are sufficient details of methods and analysis provided to allow replication by others?

Yes

Reviewer Expertise:

Primary health care, Non communicable disease control, organisation of chronic care, chronic care models, person centred care, community based models

I confirm that I have read this submission and believe that I have an appropriate level of expertise to confirm that it is of an acceptable scientific standard, however I have significant reservations, as outlined above.

# Response

**Published:** 2021-08-30

**Authors:** Joseph Mugisha Okello

**Affiliations:** Uganda Virus Research Institute, Uganda

This manuscript describes VHTs perspective on being included in the team that manages chronic conditions. While the manuscript is written clearly, there are a few methodological gaps I would like to highlight that I hope will will enable the authors to refine this report. 

1. The research question is not clear - it can be stated as an exploratory question to understand VHT experience and willingness to care for elderly with chronic conditions using a smart phone.

***Response:*** *We have addressed this in the introduction.*


*** ***

1. The methods stated are qualitative however, the sampling described is a probability sampling method - the two are not coherent. A purposive sample with a mix of men and women (if that is contextual), old and young, differing levels of education, social position, would have been appropriate to answer the question.

***Response:*** *Thank you very much for this observation. We did not use purposive sampling but as indicated, we used random sampling. As indicated, we were interested in some characteristics which we considered while selecting the sample. Even in qualitative research, random sampling can be employed depending on the context of the study.*


*** ***

1. The analysis described uses the term thematic content analysis - either this is content analysis or it is thematic.

***Response:*** *As indicated in the methods section, we used thematic content analysis to make it more clear,* *data* *were transcribed and translated (the interviews that were conducted in Luganda) into English as soon as possible after completing the interviews.* *A preliminary review for initial themes and patterns occurring within the data was done by a research assistant and the first author. The initial themes were then discussed by the two of us. Data were anonymised, coded and organised in a code matrix.  Patterns and relationships emerging across the data were established and summarised.*


*** ***

1. The analytic process described begins with themes - however in content analysis, there is no use of themes and in thematic analysis it is the result of a coding process. 

***Response:*** *Refer to my explanation in number 3 above.*


*** ***

1. The results for the experiences in dealing with older adults and knowledge of chronic conditions are stated as individual narratives however the analysis should have enabled a general theme if thematic analysis or if content analysis.

***Response:*** *Refer to my explanation in number 3 above*


*** ***

1. A table describing the participants would be useful to understand variation in response

***Response:***** *We had included this table in the first manuscript we submitted but during the review process, we were advised to remove the table by the editors.*


*** ***

1. Only practical competency for testing blood glucose and blood pressure was measured. Identification of whom to screen, when to refer and other programmatic elements including use of smartphone was not assessed. Hence to conclude that VHTs can be effectively trained is an overstatement. To conclude about effectiveness of training before and after comparison would be required.

***Response:*** *Thank you for this observation. As indicated, this was a preliminary study to prepare for a bigger study to develop and test a smart phone guided intervention to link older persons with chronic conditions to care. In this study, our objectives* *to establish the experiences of VHTs in managing older adults with health problems, their knowledge of hypertension and diabetes and their experiences with smartphones. We also investigated whether VHTs could be effectively trained to use a smartphone-guided intervention to link older adults with hypertension and diabetes mellitus to care. The identification of whom to screen and other programmatic elements will be investigated while developing and testing the smart phone guided intervention. With this, we think it was fair to come up with our conclusions.*

 We would like to thank both reviewers for their comments and we hope these will improve on the quality of our manuscript.

# Peer review: Reviewer response for version 1

**DOI:** 10.21956/aasopenres.14144.r27686

**Published:** 2020-09-02

**Authors:** Gerald A. Paccione, Kristen Welch

**Affiliations:** 1 Montefiore Medical Center, Bronx, New York, NY, USA; 2 Albert Einstein College of Medicine, New York, NY, USA

Overall, this is a scoping article that assesses VHTs’ capacity/ability to learn new skills for diagnosis of common chronic diseases (CD), and their willingness to adopt electronic aids in providing health care. Since VHTs are already part of the Ugandan health care landscape, and are only paid token amounts if anything for their community service, if the model is shown to be feasible and acceptable, scaling the training country-wide might be plausible.

 Thus, the study represents the first step in an intended multi-staged scale-up using VHTs to diagnose CDs at the community level. Community diagnosis is essential if the growing burden of downstream CD complications - like renal failure, heart failure and stroke - can be prevented before the health budgets of resource-poor countries like Uganda are drained. The ultimate goal of the project is extremely important.

 One could quibble with the need to collect and report some of the data presented in this initial-stage article, but in the scientific spirit of “assuming nothing” until numbers validate even the most basic assumptions, *the report lays a foundation for future work*. For example, many programs/projects would *assume *that VHTs - elected by their communities, deemed still active and already serving competently in maternal and child health for over 5 years (75% of study subjects), doing many VHT tasks up to now (i.e. “mobilising the communities for health action, promoting health and preventing diseases, treating simple illnesses at home, checking for danger signs at home, referring sick people to health workers and keeping up-to-date village records”) - have already *proven* that they have the cognitive skills to understand hypertension and diabetes, and can perform the most basic maneuvers like applying an *electronic* BP cuff or checking finger-stick glucose. After such an accomplished performance record, why should their lack of formal education (true in all of rural Africa), call into question their capacity for these new, fairly straightforward responsibilities? (Working as we have with Ugandan CHWs for 15 years, we’ve often thought that if they had received the early and consistent opportunities afforded Westerners or the Ugandan elite, many CHWs would be *chiefs* of medical school departments). So, if we could assume the obvious and move past it i.e. that VHTs could learn the basics of HT and DM from 3 days of lectures and training, the thrust of this first “feasibility pilot” might then have been something like investigating the ideal *duration* of training, or whether groups of available teachers from diverse district hospitals could deliver the same training intervention and realize similar success in their own catchment areas as did the highly-motivated study team in Bukulula. 

 In that vein, documenting how VHTs know that someone is “old”, or inquiring about whether the 50% of VHTs who don’t yet own a smart-phone would welcome a *free* one, seems unnecessary.

 Some specific points bear mentioning:

- The first is in the Introduction, 5 ^th^ paragraph, when reviewing similar literature to substantiate the hypothesis that the program will be successful. The studies referenced show the feasibility of using smartphone technology. Including studies that show that trained lay persons, VHTs, can be successfully taught to measure and recognize chronic diseases, and to accurately refer to health centers would certainly be pertinent. (see below for examples) ^1^^-^^4^.
- The paper covers a lot of ground, as broad as the intended (future) initiative itself: the rationale of using VHTs in chronic disease, the selection process of 20 VHTs from among the 138 eligible, an overview of the approach to the in-depth interviews (IDIs) with the 20 selected VHTs describing how the data was coded and analyzed (thematic content analysis), an overview of the training topics and practical sessions, the scheme used for evaluation of competency and it’s application/results, commentaries about VHW attitudes toward smart phones, the acceptability of using one in practice, and their (widely known) risks and challenges in impoverished settings (theft, electricity, cost).
  But it could benefit from more focus and/or more clear organization.
  It was sometimes hard to know where the paper was headed - with the themes switching rapidly, sometimes after only cursory discussion. Despite describing the IDI methodology in depth, in the end, few insightful observations or results were reported; the content of the training sessions wasn’t described beyond the titles of the topics and the credentials of who gave them; there wasn’t a (“pretest”) evaluation of what the VHWs knew about CDs before the training, or what they newly learned from the trainingconceptually beyond a technique checklist. Was the focus of the paper on exploring, through IDIs, VHTs as health care providers; VHT knowledge of CDs and ways to expand and make that knowledge functional through a 3-day curriculum; or VHT attitudes toward using smartphones in practice?
- Selection: the selection process of the VHWs could be further clarified. The methods state: *“* *The District VHT Coordinator used a raffle draw method until all the 20 VHTs were selected. The inclusion criteria were that the VHT had to be a resident of Bukulula sub-county and should have been active as a VHT within the sub-county in the last three months. VHTs were excluded if they were not in a position to participate in the interviews and were not willing to attend the training. The selection of the VHTs was done in consultation with stakeholders within the sub-county and the district including the coordinator of the* *VHTs within the district.”*
  How many times did the District VHT Coordinator draw from the raffle bin? What percent of the “draws” were excluded, and for what reason? Was it only the 2 who now lived in Kampala? The section wasn’t clear.
  Vis-à-vis VHT selection again, how representative of the entire national cohort of VHTs were the VHTs in this study is important vis-à-vis eventual scale-up? A brief discussion of the VHT program nationally, including VHT turnover and duration, would help place the description of this cohort of 20 VHTs in perspective.
- Competence testing: for BP testing, the 5 steps evaluated were named, but not described, limiting knowledge about the message communicated and its replication. How was the person “prepared for BP measurement”? What was the advice about cuff size, and about how to measure BP? Did recording accurately involve an average of measures, the first measure, the 3 ^rd^ in a series? What advice was given to the client? Mentioning the *key points* of the methods evaluated would help communicate the depth of the training.
  Also, to help future groups trying to adopt the model, it would be helpful to know more about the most common areas of difficulty in the competency tests, particularly with the 3 VHTs (15%) that failed the evaluation.
  Some of the hypertension literature suggests that it actually takes *years* of performing BP measures to do them consistently, accurately, and with minimum variability, and that electronic devices are fraught with the same variability as manual BPs in trained individuals. Time sitting prior to checking BP, back resting against a chair, arm position, talking, cuff tightness, number of BPs taken, etc. all affect the measure. Furthermore, the education literature suggests that nearly half of new knowledge learned decays after 1-3 months. With these types of clinical and educational observations in mind, lack of long-term follow-up of knowledge/skills gained through the training should be noted as a limitation of the present study, and, hopefully, if this is to be proposed as a model for CD screening, addressed by re-assessment of VHT skills in 6-12 months.
- The study makes no mention of what happens if the VHTs begin to identify the 90% of hypertensives and diabetics in Uganda who are unaware of their disease. Refer them to health centers with few staff, fewer medications, and little experience treating chronic disease? Good clinicians and health systems know that screening shouldn’t be offered for a disease if societies/families don’t have the resources to treat. So, without reliable treatment that’s affordable to the people, advocacy must be the principle objective of community-based screening. The model is not to be scaled until treatment is assured.
- The study identifies some of the barriers the VHTs foresee in using smartphones, probably stemming from their own experiences with the devices (50% of VHTs already own smart phones, indicating to these readers that the area studied is more affluent/developed than most regions of rural Uganda). These issues can probably be addressed more directly, informing the likely timing of successful rollout of the smartphone model: e.g. what percentage of the VHTs have access to electricity to charge phones and how much of an impediment does this represent in different regions of Uganda?
- Summary themes or conclusions expressed in different sections of the paper are not entirely consistent with the data presented: for example, the **title of the article** reads: *“Training village health teams (VHTs) to use a smartphone-guided intervention to link older Ugandans with hypertension and diabetes to care”.* But the article doesn’t describe training to use a smartphone-guided intervention at all. 
  In the **abstract, the objective** of the study is stated: *“we conducted this study in rural Uganda to establish the experiences of VHTs in managing older adults with health problems, their knowledge of hypertension and diabetes, and their understanding of referral systems. We also explored their experiences with smartphones and whether VHTs could be effectively trained to use a smartphone-guided intervention…” *The main objective of the article as stated here seems to be on understanding the experiences of VHTs in managing CDs in the elderly, but aside from a few highly selected anecdotes from VHTs, not much data are provided along that line. And again, there are no data presented about whether VHTs could be trained to use a smartphone intervention, aside from their willingness to do so despite potential challenges on the horizon.
  Finally, the last 4 lines of the article read: ***In conclusion*** *, VHTs in rural southwest Ugandan will need training in dealing with older people with health problems before they get involved in an intervention using a smartphone application to link older adults with chronic conditions to care. *While this is undoubtedly true - that VHTs need to know how to check BP and blood sugar to take care of adults with HTN and DM - isn’t this the premise of the training and of the report? It seems more like a “given” than a “conclusion” based on data presented in the article.
  These various summaries, conclusions and overarching objectives can be more accurately and consistently expressed.

**Conclusions:**

 Overall, this report is the first phase of an ambitious concept to utilize VHTs, previously trained in child and maternal health, in chronic disease care of adults.

 We feel it should be indexed, after addressing as many of the above limitations as possible -- many of which can be substantially remedied by tighter organization, clarification, consistency, and selective expansion of content.

Is the work clearly and accurately presented and does it cite the current literature?

Partly

If applicable, is the statistical analysis and its interpretation appropriate?

Not applicable

Are all the source data underlying the results available to ensure full reproducibility?

Partly

Is the study design appropriate and is the work technically sound?

Yes

Are the conclusions drawn adequately supported by the results?

Yes

Are sufficient details of methods and analysis provided to allow replication by others?

Partly

Reviewer Expertise:

Global Health, program implementation in Africa. General Internal Medicine.

We confirm that we have read this submission and believe that we have an appropriate level of expertise to confirm that it is of an acceptable scientific standard, however we have significant reservations, as outlined above.

## References

[1] : A pilot study to evaluate home-based screening for the common non-communicable diseases by a dedicated cadre of community health workers in a rural setting in India. *BMC Public Health* .2019;19(1) : 10.1186/s12889-018-6350-4 14 10.1186/s12889-018-6350-4 30606132PMC6318877

[2] : Detection, referral and control of diabetes and hypertension in the rural Eastern Cape Province of South Africa by community health outreach workers in the rural primary healthcare project: Health in Every Hut. *Afr J Prim Health Care Fam Med* .2018;10(1) : 10.4102/phcfm.v10i1.1610 e1-e8 10.4102/phcfm.v10i1.1610 29781685PMC5913786

[3] : An assessment of community health workers' ability to screen for cardiovascular disease risk with a simple, non-invasive risk assessment instrument in Bangladesh, Guatemala, Mexico, and South Africa: an observational study. *The Lancet Global Health* .2015;3(9) : 10.1016/S2214-109X(15)00143-6 e556-e563 10.1016/S2214-109X(15)00143-6 PMC479580726187361

[4] : Evaluation of a training program of hypertension for accredited social health activists (ASHA) in rural India. *BMC Health Services Research* .2018;18(1) : 10.1186/s12913-018-3140-8 10.1186/s12913-018-3140-8 29720161PMC5932780

# Response

**Published:** 2021-08-30

**Authors:** Joseph Mugisha Okello

**Affiliations:** Uganda Virus Research Institute, Uganda

Overall, this scoping article assesses VHTs’ capacity/ability to learn new skills for diagnosis of common chronic diseases (CD), and their willingness to adopt electronic aids in providing healthcare. Since VHTs are already part of the Ugandan health care landscape, and are only paid token amounts if anything for their community service, if the model is shown to be feasible and acceptable, scaling the training countrywide might be plausible.

 Thus, the study represents the first step in an intended multi-staged scale-up using VHTs to diagnose CDs at the community level. Community diagnosis is essential if the growing burden of downstream CD complications - like renal failure, heart failure and stroke - can be prevented before the health budgets of resource-poor countries like Uganda are drained. The ultimate goal of the project is extremely important.

 One could quibble with the need to collect and report some of the data presented in this initial stage article, but in the scientific spirit of “assuming nothing” until numbers validate even the most basic assumptions, *the report lays a foundation for future work*. For example, many programs/projects would *assume *that VHTs - elected by their communities, deemed still active and already serving competently in maternal and child health for over 5 years (75% of study subjects), doing many VHT tasks up to now (i.e. “mobilising the communities for health action, promoting health and preventing diseases, treating simple illnesses at home, checking for danger signs at home, referring sick people to health workers and keeping up-to-date village records”) – have already *proven *that they have the cognitive skills to understand hypertension and diabetes, and can perform the most basic manoeuvres like applying an *electronic *BP cuff or checking finger-stick glucose. After such an accomplished performance record, why should their lack of formal education (true in all of rural Africa), call into question their capacity for these new, straightforward responsibilities? (Working as we have with Ugandan CHWs for 15 years, we have often thought that if they had received the early and consistent opportunities afforded Westerners or the Ugandan elite, many CHWs would be *chiefs *of medical school departments). So, if we could assume the obvious and move past it i.e. that VHTs could learn the basics of HT and DM from 3 days of lectures and training, the thrust of this first “feasibility pilot” might then have been something like investigating the ideal *duration *of training, or whether groups of available teachers from diverse district hospitals could deliver the same training intervention and realize similar success in their own catchment areas as did the highly-motivated study team in Bukulula. In that vein, documenting how VHTs know that someone is “old”, or inquiring about whether the 50% of VHTs who do not yet own a smart-phone would welcome a *free *one, seems unnecessary.

**Some specific points bear mentioning:**

1. The first is in the Introduction, 5th paragraph, when reviewing similar literature to substantiate the hypothesis that the program will be successful. The studies referenced show the feasibility of using smartphone technology. Including studies that show that trained laypersons, VHTs, can be successfully taught to measure and recognize chronic diseases and to accurately refer to health centres would certainly be pertinent. (See below for examples)1-4.

***Response:*** *Thank you very much for this observation. We have added a sentence to show that studies conducted elsewhere have shown that none medical persons including community health workers can be taught to recognise chronic diseases and are able to refer those screened for chronic diseases for further management. The four references have now been cited in the introduction section.*

1. The paper covers a lot of ground, as broad as the intended (future) initiative itself: the rationale of using VHTs in chronic disease, the selection process of 20 VHTs from among the 138 eligible, an overview of the approach to the in-depth interviews (IDIs) with the 20 selected VHTs describing how the data was coded and analyzed (thematic content analysis), an overview of the training topics and practical sessions, the scheme used for evaluation of competency and it’s application/results, commentaries about VHT attitudes toward smart phones, the acceptability of using one in practice, and their (widely known) risks and challenges in impoverished settings (theft, electricity, cost). But it could benefit from more focus and/or more clear organization. It was sometimes hard to know where the paper was headed - with the themes switching rapidly, sometimes after only cursory discussion. Despite describing the IDI methodology in depth, in the end, few insightful observations or results were reported; the content of the training sessions wasn’t described beyond the titles of the topics and the credentials of who gave them; there wasn’t a (“pretest”) evaluation of what the VHWs knew about CDs before the training, or what they newly learned from the training conceptually beyond a technique checklist. Was the focus of the paper on exploring, through IDIs, VHTs as health care providers; VHT knowledge of CDs and ways to expand and make that knowledge functional through a 3-day curriculum; or VHT attitudes toward using smartphones in practice?

***Response: ***

1. Selection: the selection process of the VHWs could be further clarified. The methods state: *“The District VHT Coordinator used a raffle draw method until all the 20 VHTs were selected. The inclusion criteria were that the VHT had to be a resident of Bukulula sub-county and should have been active as a VHT within the sub-county in the last three months. VHTs were excluded if they were not in a position to participate in the interviews and were not willing to attend the training. The selection of the VHTs was done in consultation with stakeholders within the sub-county and the district including the coordinator of the VHTs within the district.” *

How many times did the District VHT Coordinator draw from the raffle bin? What percent of the “draws” were excluded, and for what reason? Was it only the 2 who now lived in Kampala? The section was not clear. Vis-à-vis VHT selection again, how representative of the entire national cohort of VHTs were the VHTs in this study is important vis-à-vis eventual scale-up? A brief discussion of the VHT program nationally, including VHT turnover and duration, would help place the description of this cohort of 20 VHTs in perspective.

***Response:*** *As we have indicated in the manuscript, the study area (Bukulula Sub County) comprises of 69 villages. Each village is supposed to have two VHTs in accordance with the Uganda Ministry of health guidelines. Thus, the total number of VHTs in this sub county were 138. As indicated, since we were going to collect qualitative data and since this was a preliminary study, 20 VHTs would give us enough data to answer our research questions.  To select the 20 from 138, a raffle method was used.  Names of all the 138 villages were written on small pieces of paper 9one paper for each village). These were put in the box and churned. The district VHTs coordinator would pick a paper and then churning would be done again. This process was repeated until 20 papers were picked. Twenty VHTs from the villages that were picked were selected to participate in the study. All the VHTs within the sub county fulfilled the inclusion criteria. Out of the 20 that were selected, only two were replaced because they stayed and worked very far away in Kampala the capital city of Uganda.*

*It is debatable on how representative were the VHTs selected for this study on the entire cohort of VHTs in Uganda. What is certain is that the guidelines for selection of VHTs in Uganda are uniform. They should have completed senior 4 (ordinary level of education in Uganda), they should be selected by members from their own communities, they should be willing to work voluntarily and should be people who understand their communities very well. we have re arranged this subsection and  added a subsection in the methods section on VHTs in Uganda.*

  

1. Competence testing: for BP testing, the 5 steps evaluated were named, but not described, limiting knowledge about the message communicated and its replication. How was the person “prepared for BP measurement”? What was the advice about cuff size, and about how to measure BP? Did recording accurately involve an average of measures, the first measure, the 3rd in a series? What advice was given to the client? Mentioning the *key points* of the methods evaluated would help communicate the depth of the training. Also, to help future groups trying to adopt the model, it would be helpful to know more about the most common areas of difficulty in the competency tests, particularly with the 3 VHTs (15%) that failed the evaluation .Some of the hypertension literature suggests that it actually takes *years *of performing BP measures to do them consistently, accurately, and with minimum variability, and that electronic devices are fraught with the same variability as manual BPs in trained individuals. Time sitting prior to checking BP, back resting against a chair, arm position, talking, cuff tightness, number of BPs taken, etc. all affect the measure. Furthermore, the education literature suggests that nearly half of new knowledge learned decays after 1-3 months. With these types of clinical and educational observations in mind, lack of long-term follow-up of knowledge/skills gained through the training should be noted as a limitation of the present study, and, hopefully, if this is to be proposed as a model for CD screening, addressed by reassessment of VHT skills in 6-12 months.

***Response:*** *Thank you for raising these important issues. We followed an SOP we use in my Unit for measuring blood pressure in older people using a digital blood pressure machine. Details on preparation of participants for BP measurements are described in the SOP. We have attached the copy of the SOP.*

  

1. The study makes no mention of what happens if the VHTs begin to identify the 90% of hypertensives and diabetics in Uganda who are unaware of their disease. Refer them to health centres with few staff, fewer medications, and little experience treating chronic disease? Good clinicians and health systems know that screening should not be offered for a disease if societies/families do not have the resources to treat. So, without reliable treatment that is affordable to the people, advocacy must be the principle objective of community based screening. The model is not to be scaled until treatment is assured.

***Response:*** *Thank you very much for this observation. You are right.  During the designing of this study, this point was considered. Originally, we had planned to pilot this intervention in people aged 35 years and above. If screening for hypertension and diabetes was done among this category and 90% of the people with hypertension and diabetes identified and referred, this would definitely overwhelm the health systems. Because of this, we decided to only consider the age category of 60 years and above. These only make up 3% of the Ugandan Population. So even if 90% of this category were screened and found to have hypertension and diabetes, these would be adequately handled by the health systems. In addition to this, all public health facilities in Uganda at the level of health centre IV (health centre at constituency level) are headed by medical doctors and stocked with hypertension and diabetes medication. These doctors would be able to handle the older people (60 years and over) screened and found with hypertension or diabetes mellitus.*

  

1. The study identifies some of the barriers the VHTs foresee in using smartphones, probably stemming from their own experiences with the devices (50% of VHTs already own smart phones, indicating to these readers that the area studied is more affluent/developed than most regions of rural Uganda). These issues can probably be addressed more directly, informing the likely timing of successful rollout of the smartphone model: e.g., what percentage of the VHTs have access to electricity to charge phones and how much of an impediment does this represent in different regions of Uganda?

***Response:*** *Thank you for this comment. The area studied is not actually affluent/developed than most of rural Uganda. Actually, smart phones are now available in most of the villages in Uganda. However, electricity coverage is not good and it is very difficult to estimate the proportion of VHTs who have access to electricity. Some village homes use solar power but it’s also not widely available.*

  

1. Summary themes or conclusions expressed in different sections of the paper are not entirely consistent with the data presented: for example, the **title of the article **reads: *“Training village health teams (VHTs) to use a smartphone-guided intervention to link older Ugandans with hypertension and diabetes to care”. *But the article does not describe training to use a smartphone-guided intervention at all. In the **abstract, the objective **of the study is stated: *“we conducted this study in rural Uganda to establish the experiences of VHTs in managing older adults with health problems, their knowledge of hypertension and diabetes, and their understanding of referral systems. We also explored their experiences with smartphones and whether VHTs could be effectively trained to use a smartphone-guided intervention…” *The main objective of the article as stated here seems to be on understanding the experiences of VHTs in managing CDs in the elderly, but aside from a few highly selected anecdotes from VHTs, not much data are provided along that line. Again, there are no data presented about whether VHTs could be trained to use a smartphone intervention, aside from their willingness to do so despite potential challenges on the horizon.

***Response:*** *Thank you very much for these observations. As indicated in the manuscript, almost all VHTs had no experiences in managing chronic conditions in older people. Almost all of the VHTs mentioned that they had only been trained to manage children with malaria, pneumonia and diarrhoeal diseases. This is the reason why we do not have data on experiences of managing chronic conditions in older persons. In addition, this is the reason we concluded that VHTs in rural southwest Ugandan will need training in dealing with older people with health problems before they get involved in an intervention using a smartphone application to link older adults with chronic conditions to care. Lastly, this study was conducted in preparation for a bigger study to develop a smart phone guided intervention. We have revised the manuscript to indicate to readers that we did not already have a smart phone guided intervention and as such, we could not have trained the VHTs on this.*

  

1. Finally, the last 4 lines of the article read: ***In conclusion*** *, VHTs in rural southwest Ugandan will need training in dealing with older people with health problems before they get involved in an intervention using a smartphone application to link older adults with chronic conditions to care. *While this is undoubtedly true - that VHTs need to know how to check BP and blood sugar to take care of adults with HTN and DM - isn’t this the premise of the training and of the report? It seems more like a “given” than a “conclusion” based on data presented in the article. These various summaries, conclusions and overarching objectives can be more accurately and consistently expressed.

***Response:*** *thank you very much. As we already indicated in response to issue 7 above, we thought VHTs would be able to describe their experiences on managing older people with chronic conditions. However, after conducting the interviews, we established that almost all the 20 VHTs had no previous experiences of managing older people with chronic conditions. Never the less, we have revised our conclusion to make it better.*

**Conclusions:**

 Overall, this report is the first phase of an ambitious concept to utilize VHTs, previously trained in child and maternal health, in chronic disease care of adults. We feel it should be indexed, after addressing as many of the above limitations as possible – many of which can be substantially remedied by tighter organization, clarification, consistency, and selective expansion of content
